# 3D computational anatomy of the scaphoid and its waist for use in fracture treatment

**DOI:** 10.1186/s13018-021-02330-8

**Published:** 2021-03-24

**Authors:** Marc-Daniel Ahrend, Teun Teunis, Hansrudi Noser, Florian Schmidutz, Geoff Richards, Boyko Gueorguiev, Lukas Kamer

**Affiliations:** 1 Department of Traumatology and Reconstructive Surgery, BG Trauma Center Tübingen, Eberhard Karls University Tübingen, Schnarrenbergstr. 95, 72076 Tübingen, Germany; 2 AO Research Institute Davos, Clavadelerstr. 8, Davos, Switzerland; 3 Plastic Surgery Department, University Medical Center Utrecht, Heidelberglaan 100, 3584 CX Utrecht, The Netherlands; 4 Department of Orthopaedic Surgery, Physical Medicine and Rehabilitation, University of Munich (LMU), Marchioninistr. 15, 81377 Munich, Germany

**Keywords:** Bone models, Anatomy of the scaphoid, Bone shape and density

## Abstract

**Background:**

A detailed understanding of scaphoid anatomy helps anatomic fracture reduction, and optimal screw position. Therefore, we analysed (1) the size and shape variations of the cartilage and osseous surface, (2) the distribution of volumetric bone mineral density (vBMD) and (3) if the vBMD values differ between a peripheral and a central screw pathway?

**Methods:**

Forty-three fresh frozen hand specimens (17 females, 26 males) were analysed with high-resolution peripheral quantitative computed tomography (HR-pQCT) and dissected to compute a 3D-statistical osseous and cartilage surface model and a 3D-averaged vBMD model of the scaphoid. 3D patterns were analysed using principal component analysis (PCA). vBMD was analysed via averaging HR-pQCT grey values and virtual bone probing along a central and peripheral pathway.

**Results:**

(1) PCA displayed most notable variation in length ranging from 1.7 cm (− 2SD) to 2.6 cm (mean) and 3.7 cm (+ 2SD) associated with differences of the width and configuration of the dorsal surface (curved and narrow (4 mm) to a wider width (9 mm)). (2) High vBMD was located in the peripheral zone. Lowest vBMD was observed in the centre and waist. (3) Virtual probing along a peripheral pathway near to the cartilage surfaces for the capitate and lunate allowed the center region to be bypassed, resulting in increased vBMD compared to a central pathway.

**Conclusion:**

High anatomical variations regarding the osseous and cartilage surfaces were associated with three distinct concentrically arranged zones with notable different vBMD. The complex scaphoid anatomy with its waist might alter the strategy of fracture fixation, education and research.

## Background

The scaphoid is the most frequently fractured carpal bone [1] and these fractures are often misdiagnosed [2]. Early diagnosis and adequate treatment are important to achieve optimal recovery and to avoid complications [3]. Unstable and displaced fractures can be stabilised by intraosseous, centrally placed screw fixation or k-wires to achieve anatomic reduction and fracture fixation [4]. The technique and approach depend on several factors such as the fracture pattern, deformity and vascularity of the proximal pole [4]. Proper fracture reduction improves outcomes and reduces the risk of radiocarpal osteoarthritis [5, 6]. However, nonunion occurs in 5% to 25% of scaphoid fractures depending on different risk factors such as smoking, displacement over 1 mm or a vertical oblique fracture line [3].

The scaphoid has a complex morphology with multiplane curvatures representing an irregular ellipsoid [7]. A detailed understanding of scaphoid anatomy helps accurate radiologic interpretation, anatomic fracture reduction and fixation. The screw or k-wire positioning is further complicated by the small waist diameter and iatrogenic cortex penetration can occur especially in small individuals [5, 8]. Also, the entry points for implant positioning are limited by several ligamentous insertions and the cartilage surface which covers 70–80% of the bone [9]. To achieve optimal positioning of the screws, a sufficiently long bone pathway and avoidance of cortex perforation is necessary [10].

Few studies have analysed the shape and size of the scaphoid in relation to possible screw or k-wire positioning [11–15]. Bone mass distribution may differ between regions of the scaphoid; however, anatomical studies and studies using clinical CT scans have provided limited insight into the distribution and optimal pathways for screw or k-wire placement and anchorage. High-resolution peripheral quantitative computed tomography (HR-pQCT) is a reliable and widely used three-dimensional (3D) imaging method for assessing bone structures and properties with a high image resolution (82 μm) [16–18].

The objective of this study was to comprehensively analyse the spatial anatomy of the scaphoid with 3D mapping of the size and shape variations of its cartilaginous and osseous surfaces and its bone mineral density distribution. We used HR-pQCT scans, dissection and 3D statistical modelling techniques to compute a 3D statistical surface model and a 3D averaged bone density model of the scaphoid. We aimed to determine (1) the size and shape variations of the cartilaginous and osseous surface of the scaphoid, (2) the volumetric bone mineral density (vBMD) distribution and (3) if the vBMD values differ between a peripheral and a central screw pathway.

## Methods

### Bone specimens

Ethical approval was not necessary. Forty-six specimens (provided by Science Care, Phoenix, Arizona, USA) gave consent for donation and use for educational and research purposes. Specimens with non-age-related bone pathologies (*n* = 2) and unreported bony alterations not known from the patient’s history (*n* = 1) were excluded. Therefore, forty-three (29 right, 14 left) fresh frozen, unpaired hand specimens were analysed. They were obtained from 17 female and 26 male donors, aged 32–93 years (mean 68.3 ± SD 15.0), with a height ranging from 135 to 203 cm (mean 169.5 ± SD 13.8 cm).

### HR-pQCT scanning

The bone specimens were thawed to room temperature and oriented in the neutral position of the wrist and with fingers extended. They were vacuum-packed and subjected to HR-pQCT imaging. After daily phantom calibration, HR-pQCT we scanned (XtremeCT™, Scanco Medical, Brüttisellen, Switzerland) of the full length of the hand specimens. The X-ray tube was set at a peak voltage of 60 kVp and 900 μA and an image matrix size of 1024 × 1024 at a nominal 82 μm isotropic voxel resolution, according to the protocol described by Kamer et al. [19]. The scanned volume of interest was adjusted on the scout view. The manufacturer’s software was used to generate the raw image data (expressed in Hounsfield units, HU) which were stored in Digital Imaging and Communications in Medicine (DICOM) format. The grey values, given in HU, were converted to vBMD values using a linear transformation defined by the machine calibration. vBMD values were expressed as milligrams of hydroxyapatite per cubic centimetre (mg HA/cm^3^). The scans were analysed with Amira, a commercially available scientific visualisation software for medical image analysis (Amira software, version 6.3.0, FEI, Hillsboro, Oregon, USA).

### HR-pQCT processing

In Amira, the HU of non-osseous tissue with HU less than 0 were set to 0. All images of the left hand were mirrored to the right. Standard threshold-based, semi-automated image segmentation was performed to create 3D computer models of each scaphoid. Nutrient foramina which could be identified due to the high scan resolution were closed manually to facilitate the subsequent data processing. HR-pQCT post-processing resulted in two types of 3D statistical models of the scaphoid: a 3D statistical surface model that demonstrated 3D size and shape and variation patterns of the bony and cartilage surfaces of the scaphoid (external configuration), and a 3D averaged bone density model which provided information about the scaphoid's internal configuration.

### 3D statistical model of osseous and cartilaginous surfaces (external configuration)

**Fig. 1:**
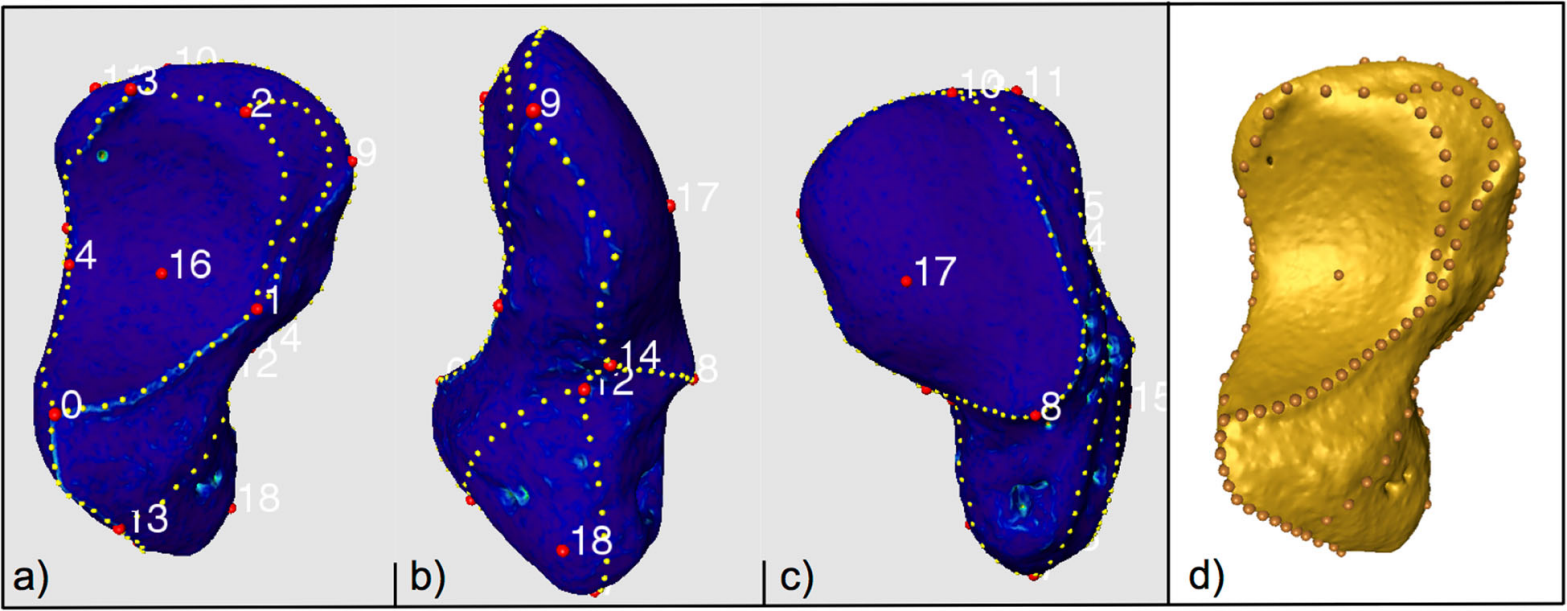
**a**–**c** Scaphoid model with manually set landmarks with ulnar view on the articular surface for capitate (**a**), volar view (**b**) and view on the articular surface for radius (**c**). **d** Scaphoid with anatomical and equidistant segment landmarks computed with view on the articular surface for capitate

Anatomical homologous landmarks and non-homologous segment landmarks, connecting them, were placed manually onto the bony surfaces of the scaphoid models (Fig. 1). Anatomical homologous landmarks represent anatomical points or bony prominences. The non-homologous segment landmarks were used in regions with sharp edges and curvatures to connect the homologous landmarks and were recomputed to obtain equally numbered and equidistant segment landmarks [20]. The cartilage areas analysed and photographed during dissection were transferred by manually setting the anatomical landmarks. All segment landmarks were recomputed to become interpolated equidistant segment landmarks.

To be able to compute homologous regions of the external configuration of the scaphoid (osseous and cartilaginous surfaces), a representative computer model with its landmark set was selected to serve as a reference model. It was warped to the remaining computer models using a thin-plate spline (TPS) transformation based on the homologous landmarks described above. TPS is an interpolation-based registration technique, which has been adapted from morphometric studies [21]. By this mean, anatomical homologous regions within all scaphoid models were established. As a result, forty-three 3D surface models of the scaphoid were computed displaying homologous osseous and cartilaginous surfaces. They were characterized by triangular meshed surfaces with identical numbers of triangles and locations (homologous 50.000 surface points and homologous 99.996 triangles).

All forty-three computer models of the scaphoid with homologous osseous and cartilaginous surfaces were aligned using a non-scaling generalised Procrustes fit algorithm [22, 23]. This computational procedure resulted in a 3D statistical model of the surface of the scaphoid including the osseous and cartilaginous surfaces.

Principal component analysis (PCA) was performed to calculate and visualise 3D size and shape variations of the scaphoid osseous and cartilaginous surfaces, as previously described [19, 23–25]. In addition, descriptive statistics for osseous and articular surfaces of the scaphoid were measured on the 3D mean model and the single models.

### 3D averaged bone density model (internal configuration)

The 3D averaged bone density model was computed as previously described for proximal humerus [19], the dens axis [26] and the pelvis [20]: It comprised homologous regions of the internal configuration of all scaphoid models. To achieve this, an isotropic reference grid with an edge length of 0.082 mm was computed within the mean model of the scaphoid. In a next step, this reference grid was transformed to the remaining scaphoids using a TPS transformation, based on homologous surface points. The corresponding vBMD values for these coordinates were calculated using trilinear interpolation. By this means, a 3D averaged bone density model of the scaphoid was created. It displays the internal configuration with grey values given in vBMD.

The bone mass distribution (vBMD) was analysed by calculating descriptive statistics for different articular, cortical, subchondral and trabecular regions of the scaphoid. In addition, two scaphoid pathways (centre pathway and peripheral pathway with optimised bone mass characteristics) were probed using Amira’s standard line probe tool with a 3 mm diameter.

## Results

### 3D surface variation

**Fig. 2:**
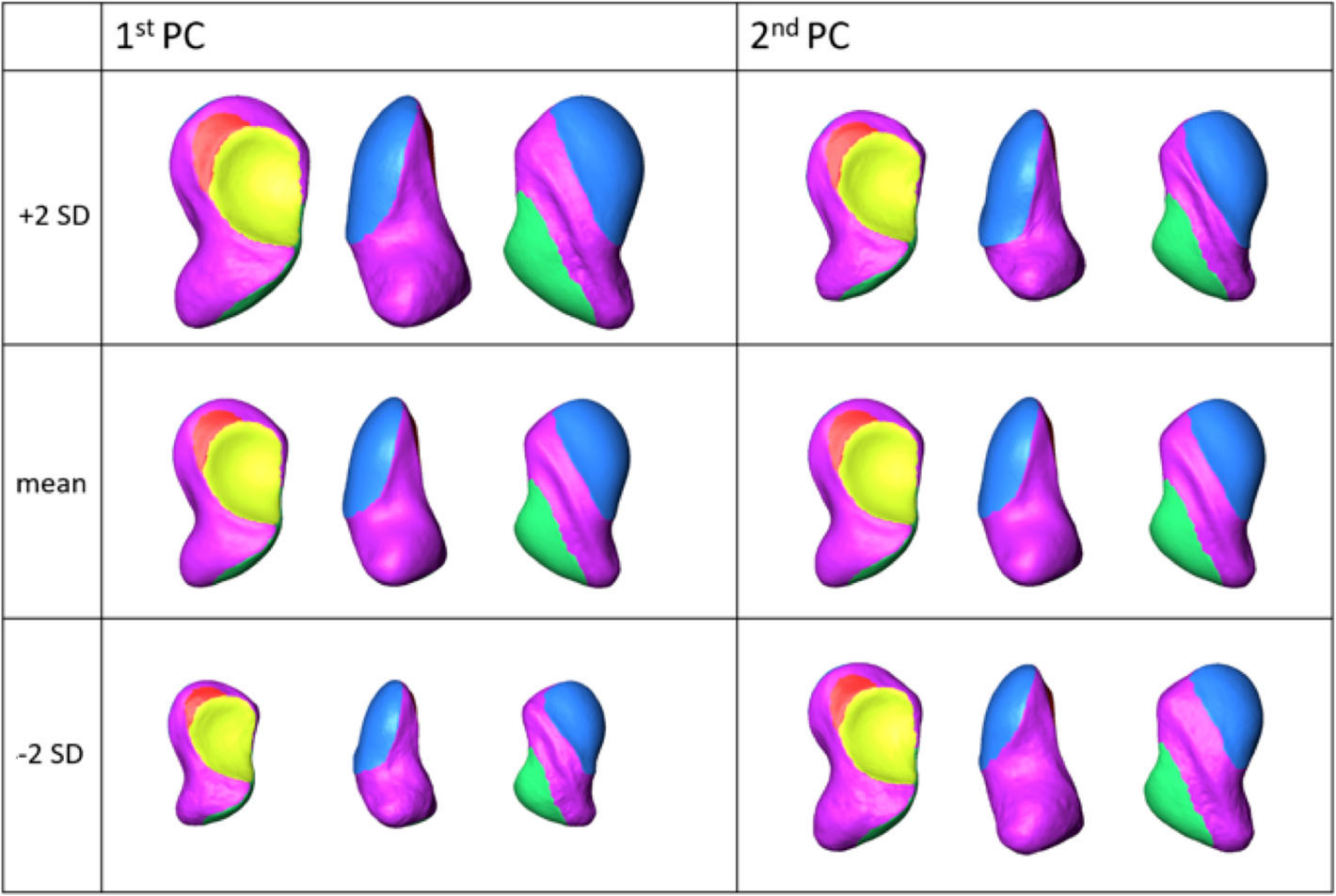
HR-pQCT-based 3D statistical surface model of the right scaphoid demonstrating 3D shape and size variation pattern of osseous and articular surfaces. Columns display 1^st^ and 2^nd^ PC with 3D views revealing most relevant variation patterns: 1^st^ PC with ± 2 SD models (top and bottom row) and mean model (middle row) mainly exhibited size variation, i.e. length. 2^nd^ PC with ± 2 SD models (top and bottom row) and mean model (middle row) with predominately a 3D shape variation of the osseous and articular surfaces (osseous surface (purple) and cartilage surfaces (yellow: cartilage surface for capitate, red: for lunate, green: for trapezium and trapezoid, blue: for radius))

PCA showed major size and shape (i.e. form) variations. The most notable variation was found in the first principal component (1^st^ PC) explaining 40.6% of the total form variation (also see Fig. 2, 1^st^ column). This 3D variation pattern was predominantly due to variation in the length of the scaphoid, ranging from 1.7 cm (− 2 SD) to 3.7 cm (+ 2 SD). Length variations in this PC were also associated with notable differences of the width and configuration of the dorsal rough surface of the scaphoid, translating from a curved and narrow (4 mm width) to a more evenly curved and wider (9 mm width) configuration (also see Fig. 2). The waist cross-section area varied with its orthogonal diameters varying from 7.8 × 8.1 mm (− 2SD) to 1.3 × 1.4 cm (+ 2D). In the second PC, shape variability was the predominant observation. The dorsal and volar sides varied from low to highly curved configurations with the osseous surfaces transforming from a narrow (− 2 SD) to a wide (+ 2 SD) configuration (also see Fig. 2, 2^nd^ column). Simultaneously, the waist translated from the centre towards the distal pole of the scaphoid. A central waist position was associated with a parallel orientation of the dorsal and volar sides; a more distal pole position of the waist markedly increased the distance between the two surfaces at the proximal pole and decreased it at the distal pole. Moreover, in the PCA model, certain variations of the 3D configurations of the articular surfaces were seen to be associated. For example, a large articular surface with the radius was associated with a narrow dorsal rough surface and vice versa. The second and the third PC accounted for an additional 10.1% and 6.5% of the total anatomical variation, thus the first three PCs made up 57.2% of the total form variation.

Descriptive statistics for the osseous and cartilaginous surfaces of the scaphoid were computed and are summarised in Table 1.

**Table 1:** Descriptive statistics for osseous and articular surfaces of the scaphoid measured on the 3D mean model and measured on the single models (mean ± SD (minimum-maximum))

Surface [mm^2^]	articular surface				Bony surface	Total
	Lunate	Capitate	Trapezium/trapezoid	Radius		
Mean: measured on 3D mean model	26.4	153.9	158.8	236.2	498.6	1073.9
Mean: measured on single models	29.9 ± 11.3 (15.2–69.6)	161.4 ± 34.9 (101.5–255.7)	166.8 ± 45.1 (92.1–280.4)	248.4 ± 61.7 (116.0–393.5)	539.7 ± 126.3 (204.3–916.0)	1146.2 ± 243.2 (663.1–1627.6)

### 3D vBMD distribution

**Fig. 3:**
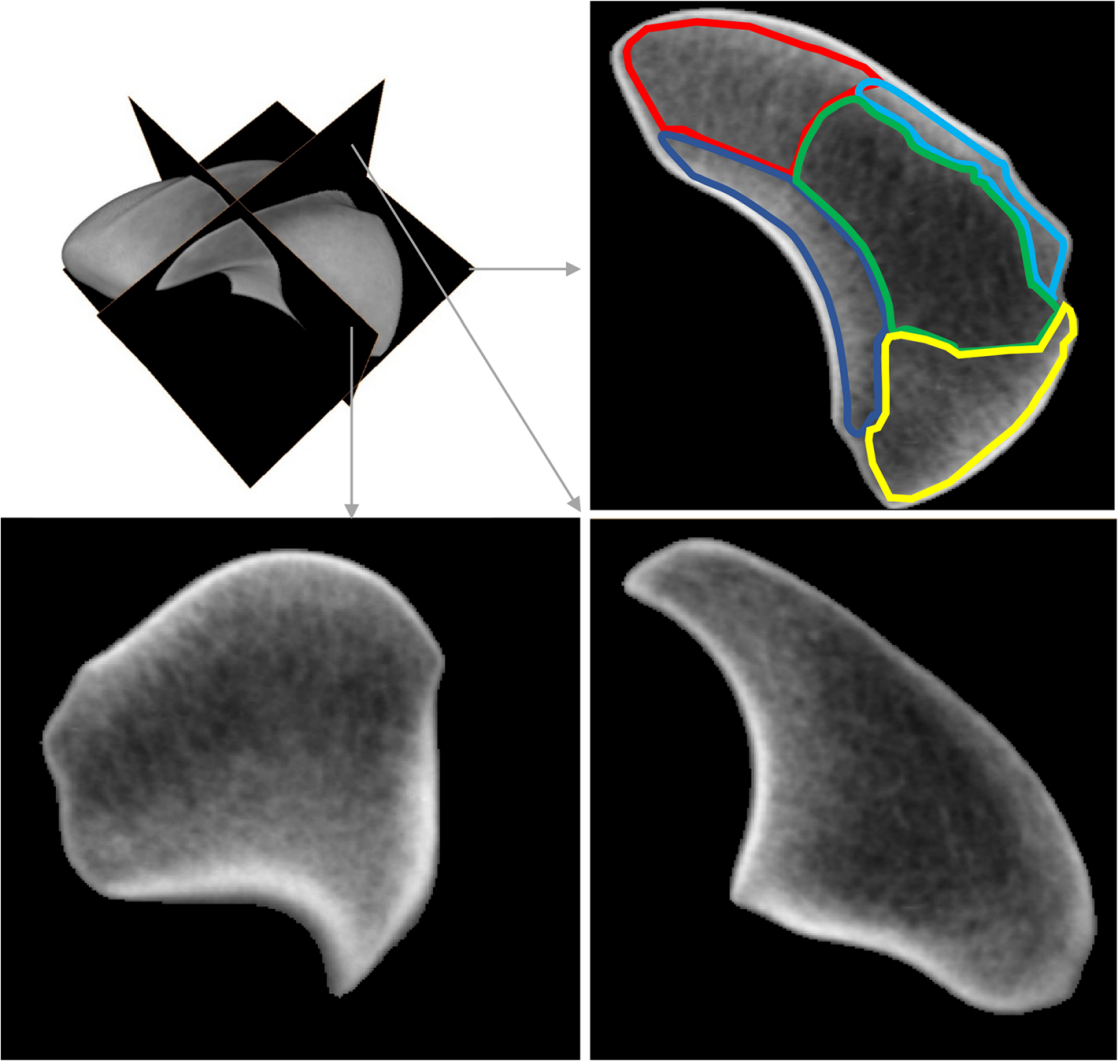
Orthogonal slice of the 3D averaged bone density model of the scaphoid including its waist. Zones with different vBMD values: outer zone (not coloured) with dense cortical and subchondral bone, intermediate zone with four subregions (light blue: lateral subregion, dark blue: medial subregion, red: proximal pole, yellow: distal pole, green: centre zone with lowest bone mass

**Fig. 4:**
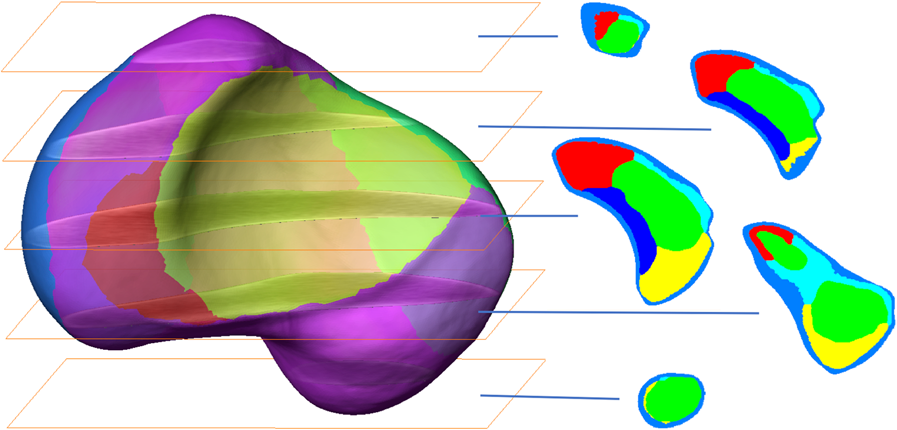
3D averaged density model of the scaphoid to characterize its inner configuration. The left figure displays the mean model of the scaphoid with osseous and cartilage surfaces. Middle and right figures exhibit orthogonal sections with the three concentrically arranged zones displaying high, intermediate and low vBMD values: 1. peripheral zone (blue) with high vBMD values; 2. intermediate zone with intermediate vBMD values and four subregions: subregion located medially (dark blue), near to the proximal pole (red); near to the distal pole (yellow) and laterally (light blue): 3. centre zone with lowest vBMD values (green)

The 3D averaged bone density model of the scaphoid displayed a specific vBMD distribution pattern. Three zones were identified, demonstrating high, intermediate and low vBMD values (Figs. 3 and 4, Table 2). High-density values were located in the peripheral zone of the scaphoid, corresponding to areas with dense cortical and subchondral bone. Inside this peripheral zone, we defined the intermediate zone, with four different subregions. Interestingly, the highest mean and maximum values were observed in the lateral subregion. The subregion near to the proximal pole demonstrated slightly higher vBMD values than the medial subregion (with articular surfaces for lunate and capitate). The subregion near to the proximal pole demonstrated about a 10% higher mean vBMD value, than the one near to the distal pole. The lowest vBMD values were observed at the centre of the scaphoid, which is where the centre of the waist was located.

**Table 2:** Descriptive statistics of the three zones and four subregions of the scaphoid (SD: Standard deviation, ccm: cubic centimetre, mgHA: milligram Hydroxyapatite)

region of interest	volume [ccm]	mean ± SD (min.-max.) [mgHA/ccm]
peripheral zone	505.8	549.4 ± 135.4 (0-853)
intermediate zone, medial subregion	219.3	376.2 ± 73.8 (160-599)
intermediate zone, subregion near to proximal pole	320.7	379.0 ± 65.9 (206-645)
intermediate zone, subregion near to distal pole	304.6	346.5 ± 84.0 (134-569)
intermediate zone, lateral subregion	172.8	389.1 ± 76.7 (153-601)
center zone	890.8	225.6 ± 63.9 (69-560)

### Virtual probing of scaphoid pathways

**Fig. 5:**
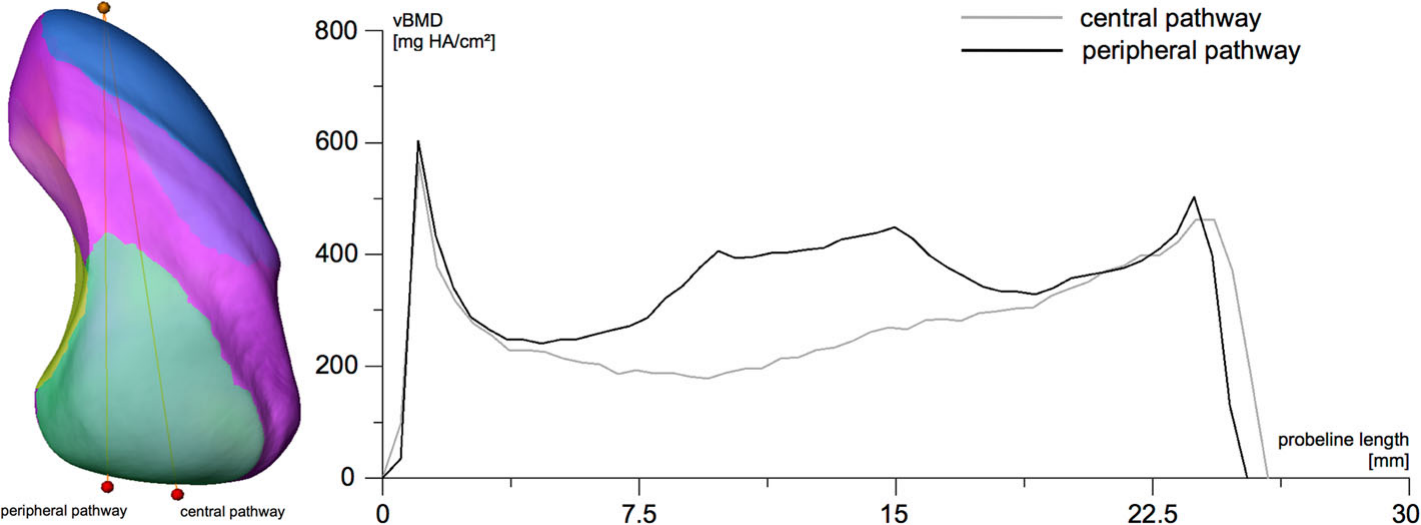
Virtual probing (3.0 mm diameter) for a central and peripheral pathway

Probing of the central pathway resulted in a line curve that included the centre zone of the scaphoid (Fig. 5, grey line). Probing along a peripheral pathway near to the cartilage surfaces for the capitate and lunate bypassed the centre region of the scaphoid (Fig. 5, black line) and had a line curve pattern with higher vBMD values compared to the centre pathway.

## Discussion

This study aimed to enhance our knowledge and understanding of the anatomy of the scaphoid, particularly its size and shape variations and bone mass distribution. This information can aid for implant development.

Our study demonstrated unexpectedly high and complex variations in size (663 to 1628 mm^2^), shape, and position of the scaphoid and its waist, transforming osseous and cartilaginous surfaces into structures with high dissimilarities. As expected, PCA showed size rather than shape to be the predominant surface variation pattern. The large surface variation pattern observed in this study might explain the different fracture patterns seen, although this is something future studies will need to assess. Surface variation is important for selecting the screw type, size and position. Scaphoid anatomy is a topic of some controversy. A study by Heinzelmann et al. [8] measured 30 scaphoid pairs with the use of a caliper. The axis from proximal pole to the distal articular surface was in male scaphoids 31.3 ± 2.1 mm and in female specimens 27.3 ± 1.7 mm. Pichler et al. [27] differed from Heinzelmann et al. [8], and similarly, we found smaller values. In men, the middle screw path was 27.1 ± 2.5 mm and in women, it was 23.3 ± 1.4 mm. Pichler et al. [27] found similar measures (27.8 ± 1.6 mm in men and in women 24.5 ± 1.6 mm). These earlier studies of the anatomy of the scaphoid analysed specimens by manually measuring the morphology with a caliper or two-dimensional slices of CT scans [8, 28, 29] which can be imprecise and disregards the complex and variable three-dimensional anatomy. Only a few studies besides ours have used 3D reconstruction of computed tomography [15, 27, 30, 31]. Van de Giessen et al. [32] calculated a statistical shape model of the scaphoid using a series of 50 CT scans with an average isotropic voxel of 0.3 mm. The HR-pQCT used in our study had a significantly higher isotropic voxel resolution (82 μm), which is, in our opinion, necessary to describe form variations in detail. Moreover, Van de Giessen et al. [32] did not include osseous and cartilaginous surface variations or the internal configuration of the scaphoid. We assessed these by computing a 3D statistical surface and a 3D averaged bone density model, using HR-pQCT scans, state-of-the-art image processing and 3D statistical modelling techniques, previously described for different skeletal sites [19, 23, 25, 33]. Moreover, surgical dissection of the scaphoid specimens was made to properly identify osseous and cartilaginous surfaces. Statistical shape modelling is a computational technique that is typically used to generate models to assess variation patterns such as bony surfaces [21, 34–36]. However, despite the advantages of 3D statistical surface models and 3D averaged bone density, they are demanding to compute and to analyse and they do not depict typical morphologic features that may be assessed in the clinical setting. Nevertheless, these models can be used to identify typical anatomical patterns, and to assess complex and variable anatomy. The approach used may be of interest also for other bones or for different research, development or teaching applications. For example, entry points and bony pathways, with or without being combined with 3D size and shape configurations, may be assessed to design new implants to improve osteosynthesis design and enhance the teaching of screw positioning in surgical training.

We found three general bone density zones, arranged spherically similar to an onion skin. The highest vBMD values were at the peripheral zones of the scaphoid, corresponding to areas with dense cortical and subchondral bone. The centre zone was found to have the largest volume, but the lowest vBMD values. Interestingly, this zone corresponded to the mid-portion of the scaphoid where the centre of the waist is located. Su-Bum et al. [37] found in CT scans of 20 male scaphoids the highest bone density parameters at regions that articulate with the radius and the capitate. The low bone density at the centre combined with the narrow anatomical shape of the waist explains the frequency of fractures at this site which is approximately 75% of all scaphoid fractures [3].

When fixing scaphoid fractures with a screw, surgeons usually aim for a centre pathway with the tip reaching as far as the opposite pole. Some authors claim that perpendicular placement to the fracture is most important, while others emphasise screw length [38–40].

A biomechanical study by Patel et al. [40] indicated that in a proximal fracture, a longer non-perpendicular screw provided more compression than a shorter perpendicular screw. In our study, vBMD values were higher at the two poles and the subchondral region to the capitate than in the centre of the scaphoid. Accordingly, virtual probing along a peripheral pathway near to the capitate and lunate articular surfaces displayed a vBMD profile with markedly increased values. We hypothesise that this would improve implant purchase and fragment compression, even with a shorter implant and might reduce nonunion or delayed union, which occurs in 5.8% of cases following screw fixation in minimally or nondisplaced fractures [41]. However, this has to be further investigated. The disadvantage of a subchondral pathway is that it potentially increases the risk of cortex perforation, which represents approximately 16% of the complications following screw fixation [41]. Moreover, we acknowledge that in clinical practice—particularly in small scaphoids—it might not be possible to select a specific pathway.

This study has several limitations. First, our results are based on a limited sample of specimens from a single population and may not be generalisable to other populations or to a clinical setting. The mean specimen’s age was 68.3 years, and the aging process could affect the shape, geometry as well as bone density [42, 43]. Secondly, the screw pathways used in this study do not account for fracture patterns and can only partially account for neighbouring carpal bones or ligament attachments. Similarly, we could not account for full wrist flexion or extension performed during surgery. The application of our findings to clinical practice, therefore, requires further study. Thirdly, we cannot account for the effect of screw placement on bony perfusion depending on different implant positioning. Future studies could assess the effect of screw or k-wire positioning on fracture union and adverse event rates and if selecting a pathway is feasible during percutaneous screw placement.

## Conclusion

In conclusion, the scaphoid size and shape vary greatly within a single population. This might explain the inconsistent anatomic descriptions [44] and perhaps be related to different fracture patterns. The scaphoid bone consists of three vBMD zones. When investigating different screw pathways, we found that a peripheral pathway along the subchondral region to the capitate might offer favourable screw purchase, allowing for more rigid fixation and reducing nonunion. Future studies can investigate if selecting a pathway is even feasible during percutaneous screw placement, and if this has an effect on union of the scaphoid.

## Data Availability

The datasets used and/or analysed during the current study are available from the corresponding author on reasonable request.

## Abbreviations

HR-pQCT: High-resolution peripheral quantitative computed tomography
PC: Principal component
PCA: Principal component analysis
SD: Standard deviation
vBMD: Volumetric bone mineral density
